# The efficacy and safety of levilimab in severely ill COVID-19 patients not requiring mechanical ventilation: results of a multicenter randomized double-blind placebo-controlled phase III CORONA clinical study

**DOI:** 10.1007/s00011-021-01507-5

**Published:** 2021-09-29

**Authors:** Nikita V. Lomakin, Bulat A. Bakirov, Denis N. Protsenko, Vadim I. Mazurov, Gaziyavdibir H. Musaev, Olga M. Moiseeva, Elena S. Pasechnik, Vladimir V. Popov, Elena A. Smolyarchuk, Ivan G. Gordeev, Mikhail Yu Gilyarov, Darya S. Fomina, Anton I. Seleznev, Yulia N. Linkova, Ekaterina A. Dokukina, Anna V. Eremeeva, Polina S. Pukhtinskaia, Maria A. Morozova, Arina V. Zinkina-Orikhan, Anton A. Lutckii

**Affiliations:** 1 Federal State Budgetary Institution Central Clinical Hospital of the Management Affair of President Russian Federation (FSBI CCH), Moscow, Russian Federation; 2 Federal State Budgetary Educational Institution of Higher Education “Bashkir State Medical University of the Ministry of Healthcare of the Russian Federation” (FSBEI HE BSMU of the Ministry of Health of Russia), Ufa, Russian Federation; 3 State Budgetary Healthcare Institution of the City of Moscow Municipal Clinical Hospital No. 40 of the Moscow Healthcare Department (SBHI MCH No. 40 MHD), Moscow, Russian Federation; 4 Federal State Budgetary Educational Institution of Higher Education “I.I. Mechnikov North-Western State Medical University”, Ministry of Healthcare of the Russian Federation (FSBEI HE I.I. Mechnikov NWSMU, Ministry of Health of Russia), St. Petersburg, Russian Federation; 5 State Budgetary Institution of the Republic of Dagestan “Republican Clinical Hospital” (SBU RD RCH), Makhachkala, Russian Federation; 6 Federal State Budgetary Institution “Almazov National Medical Research Center”, Ministry of Health of the Russian Federation (Almazov National Medical Research Center), St. Petersburg, Russian Federation; 7 State Budgetary Healthcare Institution «Kaluga Regional Clinical Hospital» (SBHI KR KRCH), Kaluga, Russian Federation; 8 Private Healthcare Institution N.A. Semashko Clinical Hospital «RZD-Medicine» (PHI N.A. Semashko Railroad Clinical Hospital), (Formerly Known As Non-State Healthcare Institution N.A. Semashko Railroad Clinical Hospital at the Lyublino Station of the JSC Russian Railways), Institute of Continues Medical Education Moscow State University of Food Production, Moscow, Russian Federation; 9 Federal State Autonomous Educational Institution of Higher Education I.M. Sechenov First Moscow State Medical University, Ministry of Health of the Russian Federation (Sechenov University), [FSAEI HE I.M. Sechenov First MSMU, Ministry of Health of Russia (Sechenov University)], Moscow, Russian Federation; 10 Moscow State Budgetary Healthcare Institution O.M. Filatov Municipal Clinical Hospital No. 15 of the Moscow Healthcare Department) (SBHI MCH No. 15 MHD) (formerly known as the State Healthcare Institution of Moscow O.M. Filatov Municipal Clinical Hospital No. 15 of the Moscow Healthcare Department), Moscow, Russian Federation; 11 State Budgetary Healthcare Institution of the City of Moscow N.I. Pirogov Municipal Clinical Hospital No. 1 of the Moscow Healthcare Department (N.I. Pirogov MCH No. 1), Moscow, Russian Federation; 12 State Budgetary Healthcare Institution of the City of Moscow Municipal Clinical Hospital No. 52 of the Moscow Healthcare Department (SBHI MCH No. 52 MHD), Moscow, Russian Federation; 13 Federal State Autonomous Educational Institution of Higher Education I.M. Sechenov First Moscow State Medical University, Ministry of Health of the Russian Federation (Sechenov University, FSAEI HE I.M. Sechenov First MSMU, Ministry of Health of Russia (Sechenov University), Moscow, Russian Federation; 14 JSC BIOCAD, Ul. Italianskaya 17, St-Petersburg, Russia 191186

**Keywords:** Levilimab, IL-6R inhibitor, COVID-19, 7-category ordinal scale

## Abstract

**Objective and design:**

The aim of this double-blind, placebo-controlled, phase III CORONA clinical trial was to evaluate the efficacy and safety of IL-6 receptor inhibitor levilimab (LVL) in subjects with severe COVID-19.

**Subjects:**

The study included 217 patients. The eligible were men and non-pregnant women aged 18 years or older, hospitalized for severe COVID-19 pneumonia.

**Treatment:**

206 subjects were randomized (1:1) to receive single subcutaneous administration of LVL 324 mg or placebo, both in combination with standard of care (SOC). 204 patients received allocated therapy. After the LVL/placebo administration in case of deterioration of symptoms, the investigator could perform a single open-label LVL 324 mg administration as the rescue therapy.

**Methods:**

The primary efficacy endpoint was the proportion of patients with sustained clinical improvement on the 7-category ordinal scale on Day 14. All efficacy data obtained after rescue therapy administration were considered missing. For primary efficacy analysis, all subjects with missing data were considered non-responders.

**Results:**

63.1% and 42.7% of patients in the LVL and in the placebo groups, respectively, achieved sustained clinical improvement on Day 14 (*P *= .0017). The frequency of adverse drug reactions was comparable between the groups.

**Conclusion:**

In patients with radiologically confirmed SARS-CoV-2 pneumonia, requiring or not oxygen therapy (but not ventilation) with no signs of other active infection administration of LVL + SOC results in an increase of sustained clinical improvement rate.

**Trail registration:**

The trial is registered at the US National Institutes of Health (ClinicalTrials.gov; NCT04397562).

## Introduction

From late 2019 when first cases of pneumonia of unknown etiology were described, the new fast spreading severe acute respiratory syndrome coronavirus 2 (SARS-CoV-2) infection (COVID-19) became the major newsmaker and a great challenge for all the humanity [1]. An extremely extensive research program provided permanently growing amount of data but still left some knowledge gaps in COVID-19 pathogenesis and immunological mechanisms of defense.

The natural course of COVID-19 in most cases present as asymptomatic or mild disease limited to replication of SARS-CoV-2 in epithelial cells of the respiratory tract which induces limited innate immune response. However, in approximately 20% of cases COVID-19 may progress to severe disease caused by the exaggerated immune response to the virus, diffuse alveolar damage, poorly controlled release of proinflammatory cytokines, development of acute respiratory distress syndrome, multiple organ failure and coagulation abnormalities [2, 3]. Increased level of proinflammatory cytokines is associated with high viral load, lung injury, disease severity and poor outcome [3–5]. Assuming that subjects with severe infection can benefit from suppression of cytokine release syndrome, a large number of clinical trials aimed to investigate the efficacy of anti-inflammatory therapy including anti-IL-1, anti-IL-6 agents and steroids in patients with COVID-19 were started [6].

Levilimab (LVL) is an original monoclonal antibody that binds to the alpha subunit of the IL-6 receptor (IL-6R) and blocks the transmission of IL-6 signal into cells. In a phase I clinical study LVL was well tolerated, showed favorable safety profile and low immunogenicity in doses ranged from 0.006 mg/kg to 2.9 mg/kg in healthy volunteers [7]. A phase II placebo-controlled clinical study showed that LVL 162 mg administered subcutaneously (SC) either once weekly (QW) or once every 2 weeks (Q2W) plus methotrexate (MTX) was superior to MTX alone in patients with active rheumatoid arthritis (RA) and inadequate response to MTX (NCT03455842) [8].

Considering the role of IL-6 pathway in the pathogenesis of severe COVID-19 and the confirmed LVL ability to block the IL-6 signaling, as well as its safety and good tolerability, we conducted a phase III clinical study to evaluate the efficacy and safety of LVL in subjects with severe COVID-19 [9].

## Patients and methods

### Study design

CORONA was a multicenter, comparative, randomized (1:1), double-blind, placebo-controlled, parallel groups phase III clinical trial conducted at 12 investigational sites in the Russian Federation in accordance with the Declaration of Helsinki and International Council for Harmonization E6 Guideline for Good Clinical Practice. The study was approved by the Central Regulatory Authority of the Russian Federation and Ethical Review Boards of each of the participating sites. The sponsor designed the trial, was responsible for the monitoring, collected the data, and performed the data analysis.

Patients or their legally authorized representatives provided written informed consent (IC) to participate in the study. If a patient was unable to give consent due to the current health status, a council of three independent physicians could make the decision to enroll the patient in the study, and the patient or his/her legally authorized representative was notified about the study as soon as possible. After the improvement in clinical status written IC was obtained.

CORONA clinical study had an adaptive design with the pre-planned opportunity to modify the endpoints, LVL doses, sample size, or the size of the study groups.

### Eligibility

Eligible were men and non-pregnant women aged 18 years or older, positive for SARS-CoV-2 RNA, hospitalized with radiologically confirmed pneumonia with at least one criteria of disease severity (respiratory rate > 30/min, SpO_2_ ≤ 93%; PaO_2_/FiO_2_ ≤ 300 mmHg; increase of the lung involvement by more than 50% after 24–48 h; decreased consciousness level; agitation; unstable hemodynamics; arterial blood lactate > 2 mmol/L; quick sequential organ failure assessment score (qSOFA) > 2, defined by the presence of any two symptoms of the following: systolic blood pressure ≤ 100 mm Hg; respiratory rate ≥ 22/min; Glasgow Coma Scale score ≤ 14).

Patients with a critical form of COVID-19 (defined by the presence of any of the following: respiratory failure and need of the invasive mechanical ventilation; septic shock; multiple organ failure); suspected active bacterial, fungal, viral, or other infection (besides COVID-19); confirmed active tuberculosis; life expectancy < 24 h, in the opinion of the investigator or who were unlikely to remain at the investigational site beyond 48 h; treated with other monoclonal antibodies, immunosuppressive agents or participating in a clinical trials of other drug; history of allergic reaction to monoclonal antibodies; who have any illness or laboratory findings that, in the opinion of the study investigator, might pose an additional risk to the patient by their participation in the study; pregnant or breastfeeding women were not eligible. Laboratory exclusion criteria were: ALT and/or AST levels > 10 × ULN, platelet count < 50 × 10^9^/L, absolute neutrophil count < 1.0 × 10^9^/L.

The use of other monoclonal antibodies for the treatment of COVID-19 was not allowed.

### Interventions

Eligible patients were stratified according to the C-reactive protein (CRP) level (CRP ≤ 7 mg/L; CRP > 7 mg/L) and then randomized at a 1:1 ratio to receive either LVL 324 mg SC (LVL group) or placebo (placebo group) on Day 1 at the investigational site. Single LVL 324 mg administration was performed as two SC injections of LVL 162 mg at a time.

During the study all patients continued to receive the standard of care therapy (SOC) in accordance with the National clinical guidelines of the Ministry of Health of the Russian Federation, which included symptomatic treatment, antiviral agents, anticoagulants, supportive care, etc.

In case of worsening of clinical status (increased SOFA score ≥ 2 and/or deterioration of respiratory or oxygenation parameters, blood pressure, vital signs, etc.) the investigator could give the subject a single open-label SC administration of LVL 324 mg as the rescue therapy.

Health status of the study subjects was assessed for the 30 days of the main study period or until the subject was discharged or until death, whichever occurred first. The follow-up telephone contact was performed on Day 60.

### Randomization and blinding

Randomization was performed centrally. After the investigator entered the eligibility screening data, the central electronic system generated a unique subject identifier (subject ID) and a unique investigational product (IP) lot number.

The investigator and patients were blinded to the treatment allocation. LVL and placebo were provided in identical primary and secondary packages with identical labels. LVL intended for use as rescue therapy was provided unblinded and was labeled “rescue therapy”.

All randomized patients received the study treatment according to the intervention they were allocated.

### Independent data monitoring committee

The Independent Data Monitoring Committee (IDMC) included three independent medical specialists. Sponsor’s representatives were not IDMC members, had no access to the blinded data, and did not participate in voting. IDMC was involved in the assessment of the risk/benefit ratio of LVL in patients with severe COVID-19.

### Outcomes

The initial primary endpoint was the overall mortality (the proportion of subjects who died in each group). Observed mortality rate in the study population was significantly lower than the assumed value. Thus, the study had not enough power to detect the difference between the groups using overall mortality. The primary efficacy endpoint was changed to the proportion of patients with sustained clinical improvement on the 7-category ordinal scale on Day 14 after the IP administration. This outcome is suggested by FDA for the assessment of clinical efficacy of therapy in COVID-19 trials. The 7-category ordinal scale includes the following categories: 1—not hospitalized/discharged, 2—hospitalized, not requiring oxygen therapy or other medical care, 3—hospitalized, not requiring oxygen therapy, requiring other medical care (related or unrelated to COVID-19), 4—hospitalized, requiring oxygen therapy, 5—hospitalized, requiring high-flow oxygen therapy or non-invasive ventilation, 6—hospitalized, requiring mechanical ventilation or extracorporeal membrane oxygenation, 7—death. Sustained clinical improvement was defined as ≥ 2-category improvement in clinical status relative to baseline on the 7-category ordinal scale or reaching the clinical status of categories 1 or 2.

The key secondary efficacy endpoint was the proportion of patients with each of the outcomes of the 7-category ordinal scale. Other secondary efficacy endpoints included: the proportion of patients transferred to the Intensive Care Unit (ICU), duration of fever (body temperature > 38 °C; for survivors) and hospitalization (for survivors) after the IP administration, change from baseline in the Erythrocyte Sedimentation Rate (ESR), CRP, and IL-6.

Safety assessment was based on the proportion of patients with the adverse drug reactions (ADRs), grade ≥ 3 ADRs, serious ADRs, frequency of systemic or opportunistic infections, grade 4 neutropenia, hypersensitivity, and injection site reactions. ADRs were reported according to the Common Terminology Criteria for Adverse Events (CTCAE) v.5.0. Adverse events (AEs) data was coded using Medical Dictionary for Regulatory Activities (MedDRA) v. 23.0.

ESR, CRP, and IL-6 levels, as well as all safety-related laboratory parameters, were measured at local clinical laboratories of trial sites using routine methods.

### Sample size

The study hypothesis was that the efficacy of LVL in combination with SOC is superior to placebo in combination with SOC. To calculate the sample size, we used the published data on the proportion of patients with sustained clinical improvement on the 7-category ordinal scale 14 days after the other IL-6R inhibitors administration [10–12]. It was estimated that at least 93 patients had to be randomized to each study group to provide 80% power to detect the treatment effect of 20% (54% vs 34%) between the LVL and the placebo groups with the superiority hypothesis (*ɛ* > 0) at a one-sided 2.5% type I error rate.

### Statistical analysis

The main efficacy analysis was performed in the population of all randomized patients (full analysis set, FAS, *n *= 206). In patients who received rescue therapy, all efficacy data obtained after the rescue therapy administration were considered missing. In the primary efficacy analysis patients with missing data were classified as non-responders. The efficacy analysis for the key secondary endpoint and other secondary endpoints was performed without missing data imputation (complete case analysis). The safety population included all randomized patients who received study therapy (*n *= 204). The safety analysis was performed using all the collected data, regardless of the use of rescue therapy (complete case analysis), in the LVL and placebo groups.

To test the hypothesis of the LVL superiority over placebo, a one-sided 97.5% Wilson confidence interval (CI) was calculated with the established superiority margin Δ = 0. *P* value for the primary endpoint was calculated for one-sided hypothesis at a statistical significance level of 0.025.

Two-sided hypothesis tests with the statistical significance level set at 0.05 were conducted for the key secondary and secondary efficacy endpoints, as well as the safety analysis.

The categorical data were compared using Pearson’s chi-squared test or Fisher’s exact test. The quantitative data was compared with Mann–Whitney test.

**Fig. 1:**
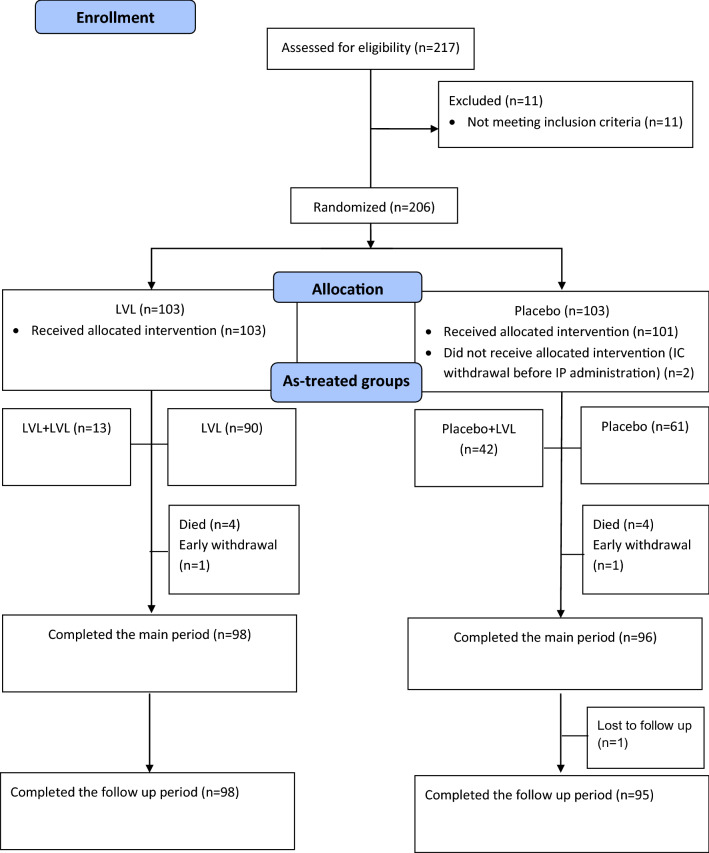
Patients flow diagram. *LVL* levilimab, *IC* informed consent, *IP* investigational product (LVL/placebo)

To confirm the validity of classifying patients who received rescue therapy as non-responders the supportive efficacy analysis was performed in as-treated groups (Fig. 1) based on the initial assignment and prescription of rescue therapy, using all collected efficacy data, regardless of the prescription of rescue therapy (without replacing the data obtained after the start of rescue therapy with missing data).

The changes of the initial clinical study plan were described in protocol amendments approved by the Central Regulatory Authority of the Russian Federation.

The statistical analysis was conducted using SAS^®^ 9.4 and the programming language R.

## Results

### Study population

The study was conducted from April 2020 to August 2020. A total of 217 patients were enrolled in the study.

Overall, 206 patients were randomized (1:1): LVL group (*n *= 103) and placebo group (*n *= 103); 2 patients in the placebo group withdrew consent before the IP administration and 204 patients received the IP (103—LVL, 101—placebo).

During the main study period 8 patients died (4 patients in each group), 2 patients were prematurely withdrawn from the study: 1 patient in the LVL group was withdrawn due to the major protocol deviation and 1 patient in the placebo group was lost to follow-up. 98 patients in the LVL group and 96 patients in the placebo group completed the main study period. During the follow-up period 1 patient in the placebo group was lost to follow-up. Thus, 98 patients in the LVL group and 95 patients in the placebo group completed the follow-up period. 13 patients in the LVL group (LVL + LVL) and 42 patients in the placebo group (placebo + LVL) received rescue therapy (Fig. 1).

### Demographic and other baseline characteristics in the FAS population

The baseline demographics, clinical characteristics and the range of concomitant diseases were almost consistent across the LVL and placebo groups. The mean age of patients was 58.5 ± 12.9 years in the LVL group and 58.2 ± 10.8 years in the placebo group, but the proportion of patients aged ≥ 75 years was higher in the LVL group than in the placebo group (11.7% (12/103) vs. 3.9% (4/103), respectively). 48.5% (50/103) and 39.8% (41/103) of patients in each group had concomitant vascular disorders and metabolism and nutrition disorders, respectively, 19.4% (20/103) of patients in the LVL group and 11.7% (12/103) of patients in the placebo group had cardiac disorders. The clinical status assessed by the 7-category ordinal scale was similar between the LVL and the placebo groups. Most patients met categories 3 and 4. The duration of fever and hospital stay were similar between the groups.

The most prescribed treatment for COVID-19 was hydroxychloroquine, anticoagulants, and antibacterials. 4.9% (5/103) of patients in the LVL group and 8.7% (9/103) of patients in the placebo group received corticosteroids (Table 1).

**Table 1:** Baseline characteristics of patients included in CORONA clinical study

Parameter	Groups “as randomized”		Groups “as treated”			
	LVL (*n *= 103)	Placebo (*n *= 103)	LVL (*n *= 90)	Placebo + LVL (*n *= 42)	LVL + LVL (*n *= 13)	Placebo (*n *= 61)
Age, years						
Mean (SD)	58.5 (12.9)	58.2 (10.8)	58.0 (11.9)	58.4 (11.7)	61.9 (18.5)	58.1 (10.3)
≥ 18 and < 40 years, *n* (%)	8 (7.8)	7 (6.8)	6 (6.7)	3 (7.1)	2 (15.4)	4 (6.6)
≥ 40 and < 65 years, *n* (%)	67 (65.1)	67 (65.1)	61 (67.8)	27 (64.3)	6 (46.2)	40 (65.6)
≥ 65 and < 75 years, *n* (%)	16 (15.5)	25 (24.3)	16 (17.8)	11 (26.2)	0	14 (23)
≥ 75 years, *n* (%)	12 (11.7)	4 (3.9)	7 (7.8)	1 (2.4)	5 (38.5)	3 (4.9)
Male gender, *n* (%)	58 (56.3)	51 (49.5)	47 (52.2)	18 (42.9)	11 (84.6)	33 (54.1)
BMI, kg/m^2^						
Median [IQR]	28.1 [25.5–31.6]	28.7 [25.5–31.6]	28.2 [25.3–31.6]	27.5 [25.1–31]	26.5 [25.9–32.3]	29.4 [26.8–32.1]
SpO_2_, %						
Median [IQR]	91.0 [90.0–92.0]	91.0 [90.0–92.0]	91.0 [90.0–92.0]	90.5 [90.0–92.0]	90.0 [89.0–92.0]	91.0 [90.0–92.0]
7-point ordinal scale category at screening, *n* (%)						
Category 5						
Hospitalized, requiring high-flow oxygen therapy or non-invasive ventilation, *n* (%)	2 (1.9)	1 (1)	1 (1.1)	0	1 (7.7)	1 (1.6)
Category 4						
Hospitalized, requiring oxygen therapy, *n* (%)	60 (58.3)	63 (61.2)	52 (57.8)	21 (50)	8 (61.5)	42 (68.9)
Category 3						
Hospitalized, not requiring oxygen therapy, requiring other medical care, *n* (%)	40 (38.8)	39 (37.9)	36 (40)	21 (50)	4 (30.8)	18 (29.5)
Category 2						
Hospitalized, not requiring oxygen therapy, not requiring other medical care, *n* (%)	1 (1)	0	1 (1.1)	0	0	0
Concomitant diseases (> 10% of patients at least in one of the groups, LVL or placebo), *n* (%)						
Vascular disorders	50 (48.5)	50 (48.5)	43 (47.8)	22 (52.4)	7 (53.9)	28 (45.9)
Metabolism and nutrition disorders	41 (39.8)	41 (39.8)	35 (38.9)	17 (40.5)	6 (46.2)	24 (39.3)
Surgical and medical procedures	19 (18.5)	17 (16.5)	17 (18.9)	9 (21.4)	2 (15.4)	8 (13.1)
Cardiac disorders	20 (19.4)	12 (11.7)	15 (16.7)	7 (16.7)	5 (38.5)	5 (8.2)
Gastrointestinal disorders	15 (14.6)	10 (9.7)	12 (13.3)	6 (14.3)	3 (23.1)	4 (6.6)
Neoplasms benign, malignant, and unspecified	13 (12.6)	8 (7.8)	9 (10)	5 (11.9)	4 (30.8)	3 (4.9)
Main disease characteristics						
Fever, *n* (%)	99 (96.1)	102 (99)	17 (18.9)	9 (21.4)	2 (15.4)	8 (13.1)
Duration of fever, days from onset to Day 1						
Median [IQR]	9 [7–13]	9 [7–13]	9 [7–13]	8 [6–11]	8 [7–12]	10 [8–14]
Duration of hospital stay, days to Day 1						
Median [IQR]	3 [2–6]	3 [2–5]	3 [2–6]	3 [2, 3]	2 [2–4]	4 [2–7]
Concomitant corticosteroids						
Dexamethasone	5 (4.9)	5 (4.9)	5 (5.6)	2 (4.8)	0	3 (4.9)
Methylprednisolone	0	4 (3.9)	0	2 (4.8)	0	2 (3.3)
Prednisolone	0	1 (1)	0	1 (2.4)	0	0
Other concomitant therapy (> 20% of patients at least in one of the groups, LVL or placebo), *n* (%)						
Hydroxychloroquine	74 (71.9)	65 (63.1)	65 (72.2)	28 (66.7)	9 (69.2)	37 (60.7)
Antithrombotic agents	70 (68)	67 (65.1)	62 (68.9)	33 (78.6)	8 (61.5)	34 (55.7)
Macrolides and lincosamides	70 (68)	67 (65.1)	60 (66.7)	33 (78.6)	10 (76.9)	34 (55.7)
Other beta-lactam antibacterials	40 (38.8)	40 (38.8)	35 (38.9)	13 (31)	5 (38.5)	27 (44.3)
Direct acting antiviral agents	27 (26.2)	25 (24.3)	26 (28.9)	11 (26.2)	1 (7.7)	14 (23)
Quinolone antibacterials	23 (22.3)	21 (20.4)	21 (23.3)	4 (9.5)	2 (15.4)	17 (27.9)
β-blocking agents	22 (21.4)	20 (19.4)	18 (20)	8 (19.1)	4 (30.8)	12 (19.7)
Other analgesics and antipyretics	19 (18.5)	23 (22.3)	18 (20)	10 (23.8)	1 (7)	13 (21.3)
Inflammatory markers at screening						
IL-6, pg/ml						
Median [IQR]	11.2 [2.7–25]	9.4 [1.7–32.2]	11 [2.5–20.9]	10.1 [4.6–32.2]	15.4 [9.7–37.7]	4.2 [1.2–34.8]
CRP, mg/l						
Median [IQR]	39.8 [20–76]	46 [18–78.4]	35.9 [20–72.5]	45.7 [18.2–80.5]	45.6 [41–76]	46 [17–76]
ESR, mm/h						
Median [IQR]	29.5 [18–45]	35 [21–50]	28 [18–45]	40 [24–51]	39 [26–48]	33 [20–50]

*LVL* levilimab, *IL-6* interleukin 6, *CRP* C-reactive protein, *ESR* Erythrocyte Sedimentation Rate

### Efficacy

#### Primary endpoint

The proportion of patients who achieved the sustained clinical improvement on Day 14 and not required rescue therapy was significantly higher in the LVL group than in the placebo group (63.1% (65/103) vs. 42.7% (44/103); *P *= 0.0017). The difference in sustained clinical improvement rate between the LVL and the placebo groups was 20.4% with one-sided 97.5% CI (7–100) (*P *= 0.0017) with its lower limit above the established superiority margin. Thus, the hypothesis of superiority of the efficacy of LVL over placebo has been confirmed.

The number of patients with sustained clinical improvement and not required rescue therapy increased in both groups throughout the main period of the study with a statistically significant predominance in the LVL group. Overall, on Day 30 84.5% (87/103) of patients in LVL group and 55.3% (57/103) of patients in placebo group met the criteria of sustained clinical improvement without rescue therapy (*P *< 0.0001).

#### Secondary endpoints

**Fig. 2:**
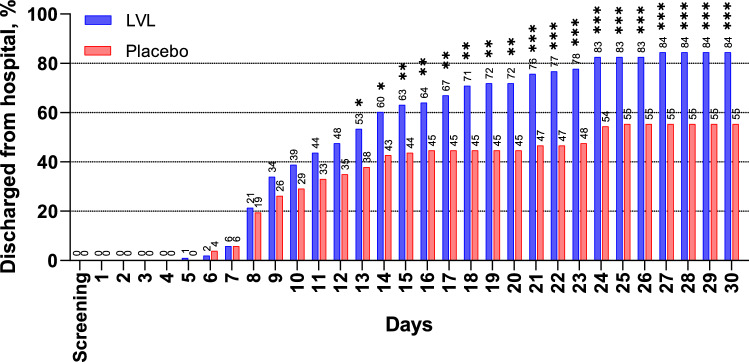
The proportion of discharged patients. *LVL* levilimab. Stars indicate the statistical significance of difference between groups as per legend. *P < .05; **P < .01; ***P < .001

From Day 1 to Day 4 there was no statistically significant difference between the LVL and the placebo groups in the number of patients meeting each of the categories of the 7-category ordinal scale and not requiring rescue therapy. On Day 5 significantly more patients treated with LVL did not require oxygen therapy (met category 3) without rescue therapy compared to the patients receiving placebo (41.8% (43/103) vs. 26.2% (27/103); *P *= 0.0186). These differences were clear until Day 12. From Day 13 and to the end of the main study period, the proportion of discharged patients (category 1) who did not require rescue therapy was significantly higher in the LVL group than in the placebo group. On Day 30, 84.5% (87/103) of patients in the LVL group and 55.3% (57/103) of patients in the placebo group were discharged (*P *< 0.0001) with no additional LVL administration (Fig. 2).

The number of patients who met categories 4–7 without rescue therapy was comparable between the LVL and the placebo groups throughout the main study period. Thus, on Day 30, none of the patients required mechanical ventilation or extracorporeal membrane oxygenation, high flow oxygen therapy or non-invasive ventilation, less than 1% (1/103) of patients in the placebo group and none of the patients in the LVL group required oxygen therapy, 1.9% (2/103) of patients in the LVL group and less than 1% (1/103) of patients in the placebo group died (*P *= 1.0000 for all the comparisons).

During the main study period fewer patients in the LVL group (3/103, 2.9%) than in the placebo group (10/103, 9.7%) were transferred to the ICU without rescue therapy (*P *= 0.0449).

After the IP administration, the duration of fever was rather short in both groups. The duration of hospital stay was also comparable between the LVL and the placebo groups. However, in patients who received rescue therapy, efficacy data obtained after the rescue therapy administration was considered missing thus the analysis of the duration of fever and hospital stay performed in subgroups (as treated) is more significant and the results are presented below.

**Fig. 3:**
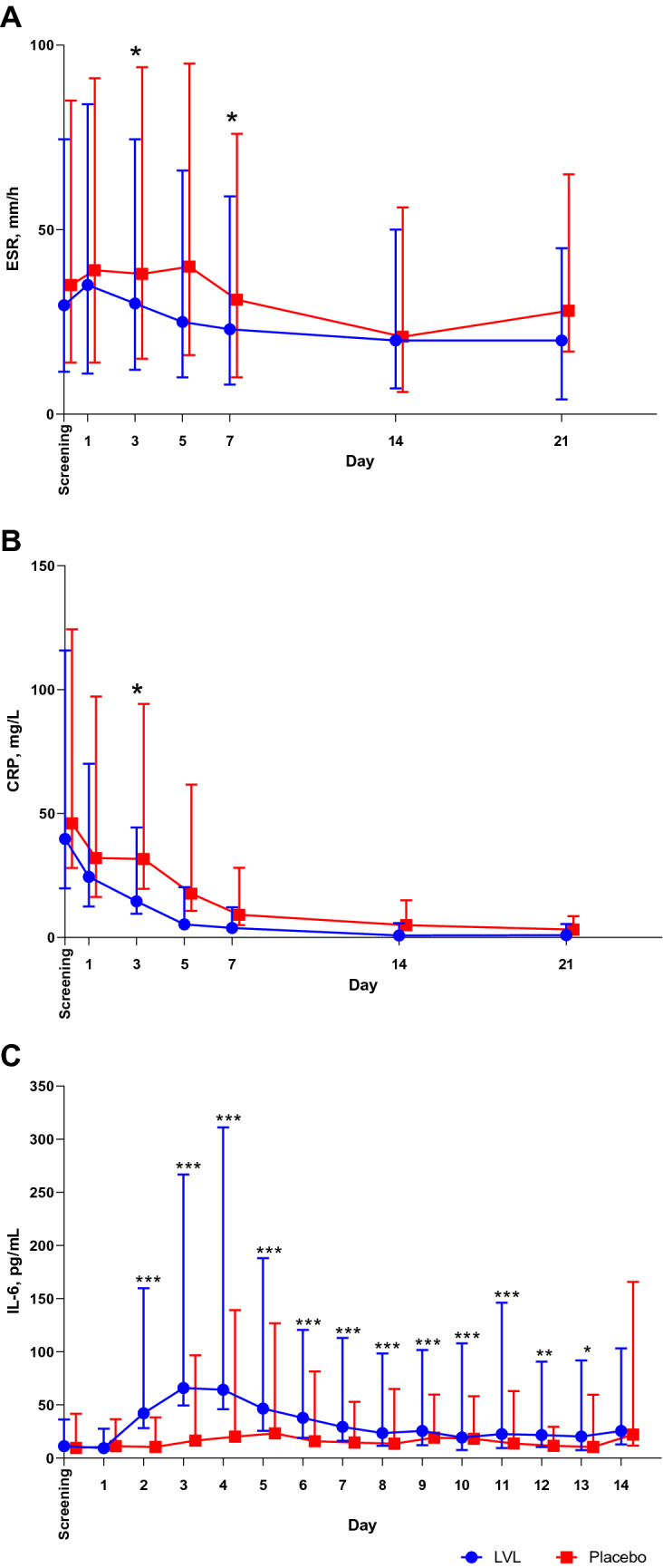
The dynamics of inflammatory markers. A. ESR, B. CRP. C. IL-6. *ESR* Erythrocyte Sedimentation Rate, *CRP* C-reactive protein, *IL-6* interleukin 6, *LVL* levilimab. Dots indicate medians and whiskers indicate upper and lower quartiles. Stars indicate the statistical significance of difference in changes from baseline of inflammation markers level between groups as per legend. *P < .05; **P < .01; ***P < .001

We evaluated the dynamics of ESR and CRP as the main inflammatory markers. After the IP administration on Day 1, the ESR distinctly decreased, but more rapidly in the LVL group: on Days 3, 5, and 7 the ESR was significantly lower in the LVL group compared to the placebo group (Fig. 3).

We observed a gradual decrease of CRP level. The CRP level was significantly lower in the LVL group compared to the placebo group on Days 3, 5 and 7 (*P *< 0.0001 for all the comparisons, Fig. 3).

The dynamics of IL-6 serum concentrations was strikingly different in the LVL and placebo groups. After the LVL administration we detected a rapid significant increase in IL-6 concentration with a slight further decline due to IL-6 inhibition representing the LVL pharmacodynamics. In the placebo group, the IL-6 concentration increased slightly until Day 2, and then decreased significantly due to clinical improvement in this group. The differences in the change of IL-6 level was statistically significant during the entire evaluation period except the Day 14 (Fig. 3).

### Supportive analysis

The number of patients who required rescue therapy due to the worsening of the clinical status was significantly greater in the placebo group compared to the LVL group: 40.8% (42/103) vs. 12.6% (13/103), respectively (*P *< 0.0001). The median time to the rescue therapy administration was 3 [IQR 2–3] days in both LVL and placebo groups (*P *= 0.7030).

On Day 14, the sustained clinical improvement rate was higher in patients who did not receive rescue therapy compared to those who received it: 72.2% (65/90), 45.2% (19/42), 53.9% (7/13), and 72.1% (44/61) of patients in the LVL, placebo + LVL, LVL + LVL, and placebo groups, respectively (*P *= 0.0105).

The differences between rescue therapy and non-rescue therapy groups were seen in the proportion of discharged patients and in the proportion of patients requiring oxygen therapy starting from Day 9.

On Day 9, the proportion of discharged patients was 38.9% (35/90) and 44.3% (27/61) in the LVL and the placebo groups and 9.5% (4/42) and 23.1% (3/13) placebo + LVL and LVL + LVL groups, respectively (*P *= 0.0006). These differences were statistically significant throughout most of the main study period.

At the same time, on Day 9, the proportion of patients requiring oxygen therapy was 38.1% (16/42) and 38.5% (5/13) in placebo + LVL and LVL + LVL groups and 16.7% (15/90) and 13.1% (8/61) in LVL and placebo groups, respectively (*P *= 0.0068). These differences remained statistically significant on Days 10, 11, 14, 15, 18.

Significantly more patients were transferred to the ICU in rescue therapy groups compared to non-rescue therapy groups: 21.4% (9/42) and 15.4% (2/13) in the placebo + LVL and LVL + LVL groups and 2.2% (2/90) and 4.9% (3/61) in the LVL and the placebo groups, respectively (*P *= 0.0006).

There was a trend to a longer fever in patients who received rescue therapy compared to patients who received the IP only, but the differences did not reach statistical significance (*P *= 0.1507). The patients who initially received LVL stayed in hospital for 3 days less than the patients who initially received placebo and required rescue therapy thereafter (*P *= 0.0391, Table 2).

**Table 2:** Efficacy of levilimab in severely ill COVID-19 patients not requiring mechanical ventilation (main secondary efficacy endpoints)

Parameter	Groups “as randomized”			Groups “as treated”			
	LVL (*n *= 103) *n* (%)	Placebo (*n *= 103) *n* (%)	*P* value	LVL (*n *= 90)	Placebo + LVL (*n *= 42)	LVL + LVL (*n *= 13)	Placebo (*n *= 61)
Patients with sustained clinical improvement, *n* (%)							
Day 7	6 (5.8)	6 (5.8)	1.0000^a^	6 (6.7)	1 (2.4)	0	6 (9.9)
Day 14	65 (63.1)	44 (42.7)	**.0017**^**b**^	65 (72.2)	19 (45.2)	7 (53.9)	44 (72.1)
Day 21	79 (76.7)	49 (47.6)	< **.0001**^**a**^	79 (87.8)	31 (73.8)	8 (61.5)	49 (80.3)
Day 28	87 (84.5)	57 (55.3)	< **.0001**^**a**^	87 (96.7)	38 (90.5)	10 (76.9)	57 (93.4)
Day 30	87 (84.5)	57 (55.3)	< **.0001**^**a**^	87 (96.7)	38 (90.5)	10 (76.9)	57 (93.4)
Patients transferred to the ICU, *n* (%)	3 (2.9)	10 (9.7)	**.0449**^**a**^	2 (2.2)	9 (21.4)	2 (15.4)	3 (4.9)
Duration of fever, days after IP administration							
Median [IQR]	1 [1–3]	2 [1–3]	.1065^c^	1 [1, 2]	2 [1–3]	1[1–3]	1 [1, 2]
Duration of hospital stay, days after IP administration							
Median [IQR]	11 [8–16]	11 [7–18]	.4852^c^	10 [7.5–15.5]	14 [10–19]	12.5 [8–21]	9 [7–13]
ESR, mm/h							
Day 3							
Median [IQR]	30 [18–44.5]	38 [23–56]	**.0035**^**c**^	29.5 [18–43.5]	45.5 [32.5–57]	30.5 [19.5–53.5]	35.5 [21–54]
Day 5							
Median [IQR]	25 [15–41]	40 [24–55]	**.0002**^**c**^	24 [15–42]	42 [27–57]	29 [16–38]	31 [21–53]
Day 7							
Median [IQR]	23.0 [15–36]	31.0 [21–45]	**.0009**^**c**^	23 [15–36.5]	34 [21–43]	27 [14–36]	30.5 [20–49]
CRP, mg/L							
Day 3							
Median [IQR]	14.6 [5.1–29.8]	31.7 [12–62.6]	** < .0001**^**c**^	12.9 [4.9–28.6]	50.8 [30–108]	28 [8.9–61]	22.4 [6–47.4]
Day 5							
Median [IQR]	5.3 [1.5–15]	17.7 [6.9–44]	** < .0001**^**c**^	5 [1.5–12.1]	22.2 [10.5–57]	8.4 [2.8–23]	14.5 [5.5–27.9]
Day 7							
Median [IQR]	3.9 [1.3–8.4]	9.2 [4.1–19]	** < .0001**^**c**^	3.8 [1.3–8.4]	9.7 [4.7–19]	4.6 [2.2–24]	8.5 [3.8–18.2]
IL-6, pg/ml							
Day 3							
Median [IQR]	65.9 [16.5–201]	16.4 [3.9–80.4]	**.0017**^**c**^	56.6 [14.8–144]	51.7 [14–163.8]	205.8 [92.4–300]	5.3 [0.7–16.7]
Day 4							
Median [IQR]	64.2 [18.3–247.1]	20.1 [1.5–119.1]	**.0121**^**c**^	48.2 [16.2–138]	78.2 [22.9–297]	300 [123.4–300]	1.5 [0.8–16.9]
Day 14							
Median [IQR]	25.4 [12.6–77.8]	108.7 [22.1–10.5]	1.0000^c^	25.1 [1.5–62.2]	33.2 [13.5–150.7]	119.2 [12.6–300]	4.6 [0.1–9.1]

*IP* investigational product, *ICU* intensive care unit, *LVL* levilimab, *IL-6* interleukin 6, *CRP* C-reactive protein, *ESR* Erythrocyte Sedimentation Rate

^a^Two-sided Pearson’s chi-squared test

^b^One-sided Pearson’s chi-squared test

^c^Mann–Whitney test

*P * < 0.05 marked in bold

### Safety

During the 60 days of the study, ADRs were reported in 27.2% (28/103) and 23.8% (24/101) of patients in the LVL and placebo groups, respectively (*P *= 0.5750) and severe ADRs (grade ≥ 3, CTCAE 5.0) were reported in 9.7% (10/103) and 6.9% (7/101) of patients in the LVL and the placebo groups, respectively (*P *= 0.4279).

Overall, 3 serious adverse events (SAEs) were reported during the study (1 in the LVL group and 2 in the placebo group); none of them were considered treatment related. In the LVL group (LVL + LVL), a 53-year-old male with a history of long-lasting hypertension and type 2 diabetes mellitus presented with unstable angina pectoris 4 days after he was discharged, he was re-hospitalized, and improved after treatment. In the placebo group (placebo + LVL), 2 patients died: a 69-year-old female with a history of chronic heart disease, atherosclerosis and type 2 diabetes mellitus presented with cardiac rhythm disturbances and a 52-year-old male progressed to respiratory failure, pulmonary thromboembolism, and respiratory distress syndrome; both deaths were considered not related to the IP in both investigator’s and Sponsor’s opinion.

6 deaths were related to the course of COVID-19 and were not reported as SAEs according to the protocol: 4 cases in the LVL group (2 cases in the LVL group and 2 cases in the LVL + LVL group) and 2 cases in the placebo group (1 case in the placebo group and 1 case in the placebo + LVL group).

Overall, 2 patients had systemic/opportunistic infections (less than 1% of patients in both the LVL and the placebo groups): 1 patient in the LVL group had grade 1 positive blood culture test and 1 patient in the placebo group had grade 2 bacteremia and grade 2 vulvovaginal candidiasis.

There were no cases of grade 4 neutropenia, hypersensitivity, or injection site reactions.

Laboratory abnormalities were the most common adverse events (AEs) during the study and were reported in 61.2% and 55.5% of patients in the LVL and the placebo groups, respectively (Table 3).

**Table 3:** Safety of levilimab in severely ill COVID-19 patients not requiring mechanical ventilation

Parameter	LVL (*n *= 103) *n* (%)	Placebo (*n *= 101) *n* (%)
Proportion of subjects with ADRs, *n* (%)	28 (27.2)	24 (23.8)
Proportion of subjects with grade ≥ 3 ADRs, *n* (%)	10 (9.7)	7 (6.9)
Proportion of subjects with serious ADRs, *n* (%)	0	0
Systemic or opportunistic infections, *n* (%)	1 (0.97)	1 (0.99)
Grade 4 neutropenia, *n* (%)	0	0
Hypersensitivity reactions, local reactions to the IP, *n* (%)	0	0
Summary of most common AEs (> 5% of patients)		
ALT increased		
Grade 1	7 (6.8)	4 (4)
Grade 2	15 (14.6)	13 (12.9)
Grade 3	11 (10.7)	6 (6)
AST increased		
Grade 1	5 (4.9)	4 (4)
Grade 2	12 (11.7)	7 (6.9)
Grade 3	7 (6.8)	4 (4)
Blood pressure increased		
Grade 2	13 (12.6)	9 (8.9)
Grade 3	9 (8.7)	10 (9.9)
Diastolic blood pressure increased		
Grade 2	17 (16.5)	13 (12.9)
Systolic blood pressure increased		
Grade 2	10 (9.7)	14 (13.9)
Grade 3	3 (2.9)	0
Neutrophil count decreased		
Grade 2	5 (4.9)	0
Grade 3	1 (1)	2 (2)
Summary of ADRs		
ALT increased		
Grade 1	3 (2.9)	2 (2)
Grade 2	13 (12.6)	10 (9.9)
Grade 3	7 (6.8)	6 (5.9)
AST increased		
Grade 1	4 (3.9)	3 (3)
Grade 2	9 (8.7)	6 (5.9)
Grade 3	7 (6.8)	4 (4)
Neutrophil count decreased		
Grade 2	2 (1.9)	0
Grade 3	0	1 (1)
Blood pressure increased		
Grade 2	0	2 (2)
Grade 3	1 (1)	0
Diastolic blood pressure increased		
Grade 2	1 (1)	1 (1)
Systolic blood pressure increased		
Grade 3	1 (1)	0
Bilirubin increased		
Grade 2	0	1 (1)
Grade 3	0	1 (1)
Lymphocytes count decreased		
Grade 2	0	1 (1)
Toxic hepatitis		
Grade 2	1 (1)	0

AE grades are presented according to the Common Terminology Criteria for Adverse Events (CTCAE) v.5.0

*ADRs* adverse drug reactions, *AEs* adverse events, *ALT* alanine aminotransferase, *AST* aspartate aminotransferase, *IP* investigational product, *LVL* levilimab

The range of ADRs was consistent with the known safety profiles of other anti-IL-6R monoclonal antibodies, as well as the safety information of levilimab obtained in the previous phase I-II clinical studies. Therefore, all ADRs were expected. No cases of early withdrawal due to safety reasons were reported.

## Discussion

During 1 year of COVID-19 pandemic multiple studies evaluating the effects of IL-6 inhibitors tocilizumab and sarilumab in COVID-19 patients showed controversial and sometimes disappointing results [4, 13–20].

The range of the placebo-controlled trials of tocilizumab failed to meet the primary efficacy endpoints such as the clinical status according to the 7-category ordinal scale within 28 days, intubation or death, number of patients requiring non-invasive or mechanical ventilation or survival [4, 13, 14, 16]. At the same time the COVACTA trial revealed the benefits of tocilizumab administration according to the other assessed outcomes: decrease of the length of the hospital stay and the decreased risk of progression to mechanical ventilation and ICU transfer. In EMPACTA trial tocilizumab administration reduced risk of start of mechanical ventilation or death [13].

Recently the REMAP-CAP trial provided new evidence of the efficacy of both tocilizumab and sarilumab based on the increased number of organ support free days and reduced mortality in critically ill ICU patients [17]. In the RECOVERY trial patients allocated to tocilizumab were more likely to be discharged from the hospital alive within 28 days and had a lower risk of invasive mechanical ventilation or death [21]. Overall, based on the results of REMAP-CAP and RECOVERY trials the use of tocilizumab and sarilumab in combination with dexamethasone was recommended in certain hospitalized patients who are exhibiting rapid respiratory decompensation due to COVID-19 [22].

It can be assumed that the diversity of the efficacy endpoints and heterogeneity of the populations with wide range of disease severity and significant differences in concomitant medication including corticosteroids could be considered the main reasons for these uncertainties.

The results of CORONA study are important and contribute to the data about the effects of anti-IL6R therapy in severely but not critically ill COVID-19 patients. Due to the ethical concerns of depriving patients in the placebo group of potentially effective treatment, open label LVL administration was used as rescue therapy in patients with worsening condition according to the pre-specified criteria. It is worth mentioning that the rescue therapy was prescribed more than 3 times less frequently in the LVL group than in the placebo group illustrating the benefit of early LVL administration.

In the CORONA study, only 5 patients in LVL group and 9 in placebo group received glucocorticoids as concomitant medication, which did not allow to perform specific subgroup analysis.

Single LVL administration in combination with SOC resulted in an increased sustained clinical improvement rate without requirement of rescue therapy in overweighted (BMI > 28) males and females, aged > 40 years old, with vascular and metabolism/nutrition disorders as main comorbidities, who have radiologically confirmed SARS-CoV-2 pneumonia with no signs of other active infection, requiring or not oxygen therapy (but not ventilation) with median saturation 91% [90.0–92.0] and increased CRP, ESR and IL-6 blood concentrations. As early as the first week after the IP administration the LVL and placebo groups were significantly different in the distribution of patients according to the ordinal scale categories. During the first 2 weeks of therapy (from Day 5 to Day 12) significantly more patients who received LVL did not need oxygen therapy compared to placebo without need of rescue therapy. After 2 weeks of therapy (IP combined with SOC) more patients were discharged in the LVL group than in the placebo group without need of rescue therapy. Patients who received LVL were transferred to the ICU 3 times less frequently than patients in the placebo group without additional LVL administration.

The supportive analysis confirmed that the rescue therapy was administered to the patients with worse course of the disease and validity of classification those who required rescue therapy as non-responders. Although the groups of patients who received and did not receive rescue therapy were similar in their demographic and clinical characteristics at screening, significantly more patients who initially received LVL and did not require rescue therapy (72.2%) demonstrated sustained clinical improvement on Day 14 than patients who initially received placebo and then required additional LVL administration (45.2%) as well as patients who received placebo.

### Overall conclusion

CORONA study had multicenter design, centrally performed randomization, the double-blinding of the allocated therapy was maintained throughout the study. The study included the homogeneous population of severely ill COVID-19 patients not requiring mechanical ventilation. The sample size was enough to test the hypothesis. IDMC was involved in the assessment of the risk/benefit ratio of LVL in patients with severe COVID-19. Thus, the risk of bias can be considered as low.

Administration of LVL + SOC resulted in an increase of sustained clinical improvement rate and decreased frequency of the ICU transfer without requirement of rescue therapy in subjects with radiologically confirmed SARS-CoV-2 pneumonia requiring or not oxygen therapy (but not ventilation) with no signs of other active infection. The rescue therapy was prescribed more frequently in the placebo group than in the LVL group suggesting the benefit of early LVL administration.

Thus, the data support that administration of LVL results in more favorable course of the disease, earlier oxygen withdrawal, and increase of sustained clinical improvement rate.

## Data Availability

The datasets generated during and analyzed during the study are available from the corresponding author on reasonable request.
